# Safety of in-hospital insertable cardiac monitor procedures performed outside the traditional settings: results from the Reveal LINQ in-office 2 international study

**DOI:** 10.1186/s12872-019-1106-3

**Published:** 2019-05-31

**Authors:** Prashanthan Sanders, Christopher Piorkowski, Johannes A. Kragten, Grahame K. Goode, Satish R. Raj, Trang Dinh, M. Rizwan Sohail, Rishi Anand, Angel Moya-Mitjans, Noreli Franco, Kurt Stromberg, John D. Rogers

**Affiliations:** 1 Department of Cardiology, Centre for Heart Rhythm Disorders, South Australian Health and Medical Research Institute, University of Adelaide and Royal Adelaide Hospital, Adelaide, SA 5000 Australia; 2 Herzzentrum Dresden- Abteilung für Invasive Elektrophysiologie, Dresden, Germany; 3 Zuyderland Medisch Centrum Heerlen, Heerlen, Netherlands; 4 Blackpool, Fylde and Wyre Hospitals, NHS Foundation, Blackpool, UK; 5 Libin Cardiovascular Institute of Alberta, University of Calgary, Calgary, Canada; 6 Maastricht University Medical Center, Maastricht, Netherlands; 7 Mayo Clinic College of Medicine and Science, Rochester, MN USA; 8 Holy Cross Hospital, Fort Lauderdale, FL USA; 9 Hospital Universitari Vall d’Hebron, Barcelona, Spain; 10 Cardiac Rhythm and Heart Failure, Medtronic, Inc, Mounds View, MN USA; 11 Scripps Prebys Cardiovascular Institute, La Jolla, CA USA

**Keywords:** Insertable cardiac monitor, Safety, Procedure room, Holding area, Duration, Resources

## Abstract

**Background:**

Historically, the majority of insertable cardiac monitor (ICM) procedures were performed in the cardiac catheterization (cath) lab, electrophysiology (EP) lab, or operating room (OR). The miniaturization of ICMs allows the procedure to be relocated within the hospital without compromising patient safety. We sought to estimate the rate of untoward events associated with procedures performed within the hospital but outside the traditional settings and to characterize resource utilization, procedure time intervals, and physician experience.

**Methods:**

The Reveal LINQ in-Office 2 (RIO 2) International study was a single arm, multicenter, prospective study. Patients indicated for an ICM and willing to undergo device insertion outside the cath/EP lab or OR were eligible and followed for 90 days after insertion.

**Results:**

A total of 191 patients (45.5% female aged 63.8 ± 26.9 years) underwent successful Reveal LINQ ICM insertion at 17 centers in Europe, Canada and Australia. The median total visit duration was 106 min (interquartile range [IQR]: 55–61). Patient preparation and patient education accounted for 10 min (IQR: 5–20) and 10 min (IQR: 8–15) of total visit duration, respectively. Preparation and education occurred in the procedure room for 90.6 and 60.2% of patients, respectively. There were no untoward events (0.0, 95% CI: 0.0–2.1%) though four patients presented with procedure-related adverse events that did not require invasive intervention. Physicians rated procedure location as convenient or very convenient.

**Conclusions:**

The Reveal LINQ™ ICM insertion can be safely and efficiently performed in the hospital outside the cath/EP lab or OR.

**Trial registration:**

ClinicalTrials.gov identifier NCT02412488; registered on April 9, 2015.

**Electronic supplementary material:**

The online version of this article (10.1186/s12872-019-1106-3) contains supplementary material, which is available to authorized users.

## Background

Subcutaneous insertable cardiac monitors (ICMs) continuously record ECG for up to 3 years and are used clinically for the detection of infrequent arrhythmias and atrial fibrillation management. The traditional settings for ICM insertion procedures have been the cardiac catheterization (cath) lab, the electrophysiology (EP) lab and the operating room (OR) - the same facilities used for more complex cardiac device implants, such as pacemakers, cardiac resynchronization therapy devices, and implantable cardioverter defibrillators. This practice became widespread due to the larger size of early ICM models. However, miniaturization of ICM devices has resulted in a less invasive insertion procedure, enabling relocation of device insertion within the hospital to clean rooms, procedure rooms and holding areas, and to practice offices outside the hospital walls [1]. This approach is appealing because it could reduce costs related to the procedure and increase physician and patient convenience and satisfaction [2, 3].

To further evaluate the safety of Reveal LINQ ICM insertion within the hospital, but outside the traditional settings, we built upon previous literature to conduct the first prospective, international multi-site trial to assess the rate of adverse events through 3 months post device insertion. The study was mainly conducted in Europe and collected patient, physician, and detailed staff procedure time interval data to document the resources necessary for the procedure.

## Methods

### Study design

The Reveal LINQ™ In-Office 2 (RIO 2) International study (NCT02412488) was a single arm, multicenter, interventional post-market study. Patients at least 18 years of age who were indicated for continuous arrhythmia monitoring with an ICM, and who were willing to undergo ICM insertion in the hospital, but outside the cath/EP lab or OR were enrolled between September 2015 and May 2016.

Patients were followed for 90 days after ICM insertion with scheduled in-person visits at days 30 and 90, and unscheduled visits as needed. At each follow-up, the patient and insertion site were evaluated, and device and/or procedure related adverse events were documented.

The primary endpoint was the occurrence of untoward events, defined as a composite of unsuccessful ICM insertion and complications related to the Reveal LINQ ICM and/or insertion procedure. Complication was defined as an adverse event (AE) resulting in death, involving termination of significant device function, or requiring invasive intervention. Infection was categorized as deep incision site or superficial. Deep incision site infections were defined as pain, redness or drainage at the incision site that required the device to be removed or IV antibiotics to be administered. Superficial infections were characterized by redness beyond procedure expectation and administration of oral antibiotics. An independent Clinical Events Committee comprised of electrophysiologists and infection specialists who were not study investigators determined whether each event met the primary endpoint.

Ancillary objectives included summarizing resource utilization, procedure duration, physician experience, and all device and procedure related adverse events, regardless of severity.

### Ethics and consent to participate

All patients provided written informed consent to the study protocol that was approved by the Human Research Ethics Committee of each participating institution.

### Insertion procedure

ICM insertion (Reveal LINQ™, model LINQ11, Medtronic, Inc.) was performed in accordance with the manufacturer’s instructions. Device insertion took place in a hospital location that was outside the cath/EP lab or OR such as a procedure room, holding area or office. Throughout the manuscript, these locations are referred to as “out-of-lab”. Physicians were required to use the incision and insertion tools, and to insert the device at one of the anatomical locations listed in the Instructions for Use. The protocol also mandated that physicians wear sterile gloves, gown and mask during device insertion, and use hand antiseptic prior to the procedure. Patients were required to be draped or wear a mask during the procedure. Sedation was not allowed, but local anesthesia and anxiolytic medications were permitted and used in accordance with physician and patient preference. Use of prophylactic antibiotics was left to physician discretion and institution specific infection control protocols. The incision was closed using adhesive strips, surgical glue, sutures or staples.

### Procedure duration and resource utilization

Procedure time intervals, procedural details, supply use, and staffing resources were recorded during pre-insertion preparation, and post-insertion activities. The time intervals characterized were: (1) Visit duration: time from patient check-in to patient discharge; (2) Process time: time from start of patient preparation to skin closure; (3) Procedure room time: time from when the patient enters the procedure room to when the patient leaves the procedure room; (4) Patient preparation: time from the start of the patient preparation to the completion of patient preparation (including the time required for clinical assessment, changing clothes and surgical site preparation); (5) Education time: time required to educate the patient with respect to their LINQ system and their incision site.

### Physician questionnaire

Physicians were required to complete a questionnaire following each ICM insertion procedure that assessed subject response, procedure delays, and physician satisfaction. See Additional file 1 for a listing of specific questions asked.

### Sample size and statistical analysis

Based on previous studies, it was anticipated that: 1) there would be no failed device insertion attempts, 2) the rate of untoward events would be similar (roughly 2%) when device insertion occurred inside or outside the cath/EP lab or OR setting, and 3) the attrition rate would be 10%. A sample size of 204 patients was computed based on the exact method to provide a target one-sided upper 95% confidence boundary within 3% of a point estimate based on the above assumptions. Additionally, a sample size of 204 patients provided an 87% chance to detect a rare LINQ™ insertion related complication occurring at a true underlying rate of 1%, and a 64% chance to detect a rare complication occurring at a true underlying rate of 0.5%.

The exact binomial method was used to construct a 95% two-sided confidence interval for the untoward event rate. Patients were considered evaluable for the primary endpoint if they had an event meeting the primary endpoint, completed the 90-day in-person visit, or had their ICM explanted during the follow-up period.

## Results

### Study population

**Fig. 1:**
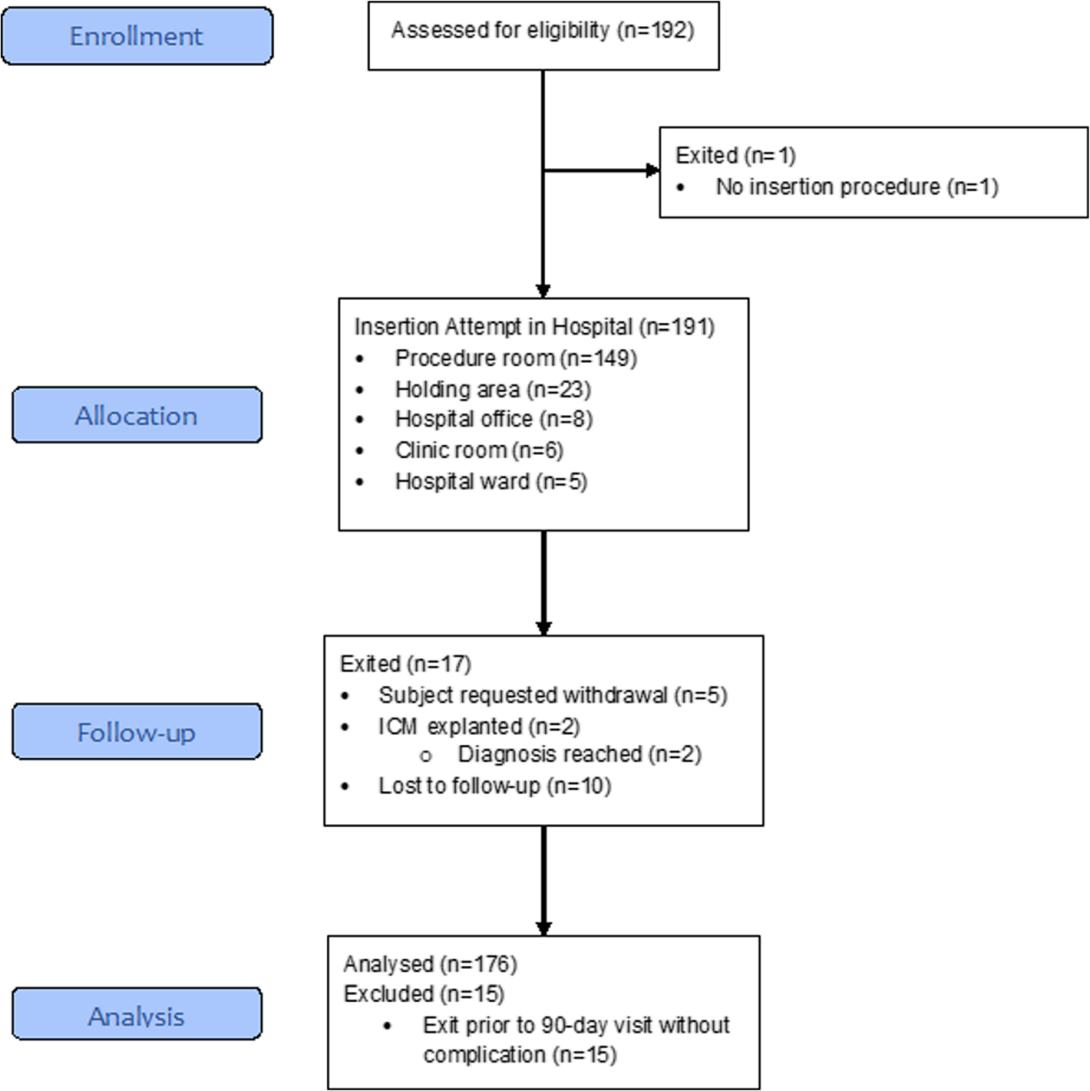
Enrollment and follow-up of study participants

The study enrolled 192 patients at 12 centers in Europe [Germany (3), Italy (3), the Netherlands (2), Spain (1), Sweden (1), and the United Kingdom (2)], 3 centers in Canada, and 2 centers in Australia between September 2015 and May 2016. Enrollment was ended early (prior to reaching the target sample size of 204) due to a lower than expected attrition rate between the time of enrollment and device insertion. Of the 192 patients enrolled, 191 underwent an ICM insertion attempt out-of-lab. All 191 insertion attempts were successful. Enrollment and follow-up of study participants is summarized in Fig. 1. Mean follow up was 3.3 ± 0.7 months, with 98% compliance to study visits.

Patient characteristics and the primary indication for an ICM device are displayed in Table 1. Of the 191 patients with an ICM insertion, 45.5% were female. The most common indication was syncope (53.9%), followed by cryptogenic stroke or TIA (17.3%).

**Table 1:** Baseline patient characteristics and ICM indication

Characteristic	Total (*N* = 191)
Sex n (%)	
Male	104 (54.5%)
Female	87 (45.5%)
Physical Exam Findings Mean ± Standard Deviation	
Age (years)	63.8 ± 26.9
Body mass index (kg/m^2^)	27.9 ± 4.8
Systolic blood pressure (mm Hg)	138.2 ± 21.6
Diastolic blood pressure (mm Hg)	78.6 ± 10.6
Primary Indication n (%)	
Atrial fibrillation ablation monitoring	3 (1.6%)
Atrial fibrillation management	18 (9.4%)
Cryptogenic stroke or TIA	33 (17.3%)
Palpitations	7 (3.7%)
Suspected atrial fibrillation	15 (7.9%)
Syncope	103 (53.9%)
Ventricular tachycardia	2 (1.0%)
Other indication	10 (5.2%)

### Procedural details

Most device insertions took place in a procedure room (78%) followed by a holding area (12%, Fig. 1). Antibiotics were administered in 44.0% of patients prior to ICM insertion. In all cases, antibiotics were administered intravenously. Local anesthesia was used in 100% of insertion procedures, whereas anxiolytic use only occurred in one patient (0.5%). Most patients were draped during device insertion (80.6%), and almost all physicians wore a mask (99.5%), gown (89.5%), and surgical head covering (90.6%). Despite some deviations from the established protocol during patient preparation, no apparent increase in the risk of adverse events was established. For 88.5% of procedures, no device repositioning was required. When repositioning was necessary (11.5% of procedures), one attempt was sufficient to achieve adequate sensing. The device was inserted 45 degrees relative to the sternum over the 4th intercostal space in 93.7% of patients. Adhesive strips were the most common closure method used (64.9% of patients) followed by sutures (28.3%, Table 2). Post-procedure antibiotic use was uncommon (1.6%); when used, it was administrated intravenously.

**Table 2:** ICM insertion procedure details

Procedure Element	Patients (*N* = 191)
Surgical site preparation^a^	
None	13 (6.8%)
Shaved with razor	50 (26.2%)
Clipped	16 (8.4%)
ChloraPrep (chlorohexidine gluconate solution)	85 (44.5%)
Providone-iodine	107 (56.0%)
Other	11 (5.8%)
Patient preparation^a^	
None	0 (0.0%)
Mask	89 (46.6%)
Gown	78 (40.8%)
Drape	154 (80.6%)
Surgical head covering	59 (30.9%)
Insertion physician preparation^a^	
None	0 (0.0%)
Surgical hand antiseptic in the room	69 (36.1%)
Surgical hand antiseptic outside the room	34 (17.8%)
Traditional surgical scrub in the room	48 (25.1%)
Traditional surgical scrub outside the room	72 (37.7%)
Insertion physician preparation materials^a^	
None	0 (0.0%)
Mask	190 (99.5%)
Gown	171 (89.5%)
Surgical head covering	173 (90.6%)
Single layer of gloves	134 (70.2%)
Double layer of gloves	57 (29.8%)
Other	7 (3.7%)
Did patient preparation take place in the procedure room?	
No	18 (9.4%)
Yes	173 (90.6%)
Location of inserted device	
45-degree relative to sternum over 4th intercostal space	179 (93.7%)
Parallel to sternum over 4th intercostal space	11 (5.8%)
Inframammary fold	0 (0.0%)
V1 ECG location	0 (0.0%)
V2 ECG location	0 (0.0%)
Other location	1 (0.5%)
Number of repositioning/modifications during procedure	
0	169 (88.5%)
1	22 (11.5%)
2	0 (0.0%)
Closure Method^a^	
No closure method used	0 (0.0%)
Sutures	54 (28.3%)
Adhesive strips	124 (64.9%)
Surgical glue	18 (9.4%)
Staple(s)	0 (0.0%)
Location of post-procedure education	
Same room as procedure	115 (60.2%)
Dedicated recovery room	59 (30.9%)
Exam room	1 (0.5%)
Waiting area	3 (1.6%)
Other location	13 (6.8%)

^a^Categories are not mutually exclusive and may sum to more than 100%

### Procedure time intervals

**Fig. 2:**
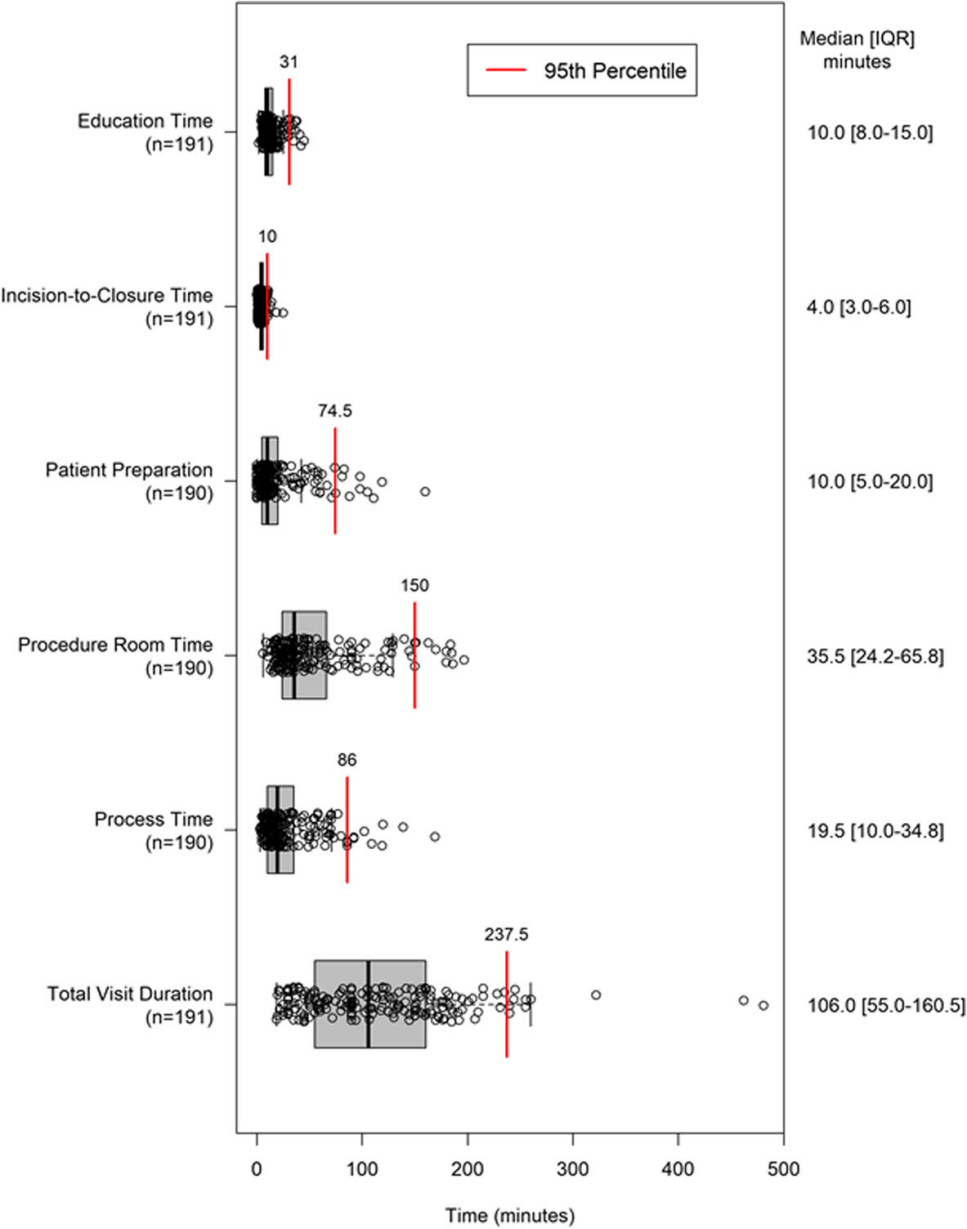
Distribution of procedure time intervals. Visit duration is the time from patient check-in to discharge. Process duration is the time from the start of patient preparation to completion of the insertion procedure (skin closure). Procedure room occupancy is the time from when the patient enters the procedure room to the time the patient leaves the procedure room. Patient preparation time is the time from the start of patient preparation to the time patient preparation is completed. Education duration is the total time required to educate the patient on the use of their ICM device and care of their insertion wound. The left and right box dimensions are the interquartile (IQR) range. Open circles represent individual patients

The total visit duration (time from check-in to discharge) was 115.4 ± 73.6 min [median: 106.0, IQR: 55–161 min]. Patient preparation accounted for 19.6 ± 25.6 min [median: 10.0 IQR: 5–20 min] and patient education accounted for 13.1 ± 7.9 min [median: 10.0, IQR: 8–15 min] of the overall visit duration on average. The distribution of procedure time intervals is displayed in Fig. 2. Patient preparation and patient education took place in the procedure room for 90.6 and 60.2% of patients, respectively. Procedure room time was 54.1 ± 43.1 min [median: 35.5, IQR: 24–66 min].

### Staffing resources

Staffing resources during pre-insertion, device insertion, and post-insertion activities are displayed in Table 3. The predominant staffing resources for pre-insertion activities included registered nurses (68.1% of procedures) and physicians (58.6% of procedures). Physicians also participated in the majority (82.2%) of device insertions. Cardiovascular/surgical technologists and physicians most commonly completed post-insertion activities (52.9 and 36.1% of procedures, respectively). The mean physician time was 10.4 ± 9.6 [median: 9.0, IQR: 5–13] minutes for pre-insertion activities, and 12.6 ± 4.8 [median: 14.0, IQR: 10–15] minutes for post-insertion activities when physicians were involved in these aspects of the procedure.

**Table 3:** Staffing resources for ICM insertion

Staffing Resource	Pre-Insertion n (%)	Device Insertion n (%)	Post-Insertion n (%)
Physician	112 (58.6%)	157 (82.2%)	69 (36.1%)
Physician assistant	48 (25.1%)	46 (24.1%)	46 (24.1%)
Nurse practitioner	22 (11.5%)	18 (9.4%)	28 (14.7%)
Registered nurse	130 (68.1%)	107 (56.0%)	43 (22.5%)
CV/OR technologist^a^	52 (27.2%)	54 (28.3%)	101 (52.9%)
Medical assistant	11 (5.8%)	5 (2.6%)	4 (2.1%)
Other	0 (0.0%)	0 (0.0%)	1 (0.5%)

^a^ Cardiovascular/surgical technologist

### Primary objective

Of the 191 patients who received an ICM, 15 were excluded from the primary analysis due to premature exit (10 lost to-follow-up, 5 requested withdrawal from the study) without experiencing an untoward event. Among the 176 patients evaluable for the primary objective there were no untoward events. Thus, the rate of untoward events was 0% with a 95% confidence interval ranging from 0 to 2.1%. A missing data sensitivity analysis indicated that patients excluded from the analysis cohort were extremely unlikely to have had an untoward event.

### ICM related adverse events

Of the 191 patients with an ICM insertion, there were four device and/or procedure related adverse events in four patients (Table 4); none met the primary endpoint definition. One of the incision site hemorrhage events was resolved when the dressing was changed; the patient was given oral acetaminophen. At the time of the procedure, the patient was taking on antiplatelet therapy. The other incision site hemorrhage event was resolved by applying pressure; the patient was on oral anticoagulation therapy at the time of the procedure. One patient experienced presyncope following incision closure due to pain during the procedure, despite administration of an extra dose of lidocaine. The patient’s blood pressure was normalized within minutes by tilt/raising the procedure bed and by giving the patient several glasses of water. Finally, no further actions were taken for the implant site pain event. There were no migrations, infections or complications related to the LINQ system or LINQ insertion procedure observed during the study.

**Table 4:** Device and/or procedure related adverse events

Device/procedure related events	Adverse Events	Complications
Implant site pain	1 (1, 0.52%)	0 (0, 0%)
Incision site hemorrhage	2 (2, 1.05%)	0 (0, 0%)
Presyncope	1 (1, 0.52%)	0 (0, 0%)
TOTAL	4 (4, 2.09%)	0 (0, 0%)

### Physician questionnaire

Responses to the physician questionnaire are displayed in Table 5. Physicians reported that 85.9% of procedures were not delayed over 15 min. Most physicians (90.6%) found the procedure room facility to be convenient or very convenient, and all physicians were satisfied or very satisfied with the insertion environment. Physicians perceived patients to respond well to device insertion, with 71.2% reporting patients having a very positive response, and 28.8% reporting patients having a positive response.

**Table 5:** Physician questionnaire responses

Questionnaire Item	Surveys (*N* = 191)
How well did subject respond to LINQ insertion n (%)	
Very positive	136 (71.2%)
Positive	55 (28.8%)
Neutral	0 (0.0%)
Negative	0 (0.0%)
Very negative	0 (0.0%)
No response	0 (0.0%)
Procedure delay > 15 min n (%)	
Yes	27 (14.1%)
No	164 (85.9%)
No Response	0 (0.0%)
Estimated physician work time during “pre-service period” (minutes)	
Mean ± Standard Deviation	14.9 ± 14.3
Median [Interquartile Range]	10.0 [5.0–15.0]
Estimated physician work time during “post-service period” (minutes)	
Mean ± Standard Deviation	14.1 ± 10.9
Median [Interquartile Range]	15.0 [5.0–15.0]
Convenience of facility n (%)	
Very convenient	136 (71.2%)
Convenient	37 (19.4%)
About the same	18 (9.4%)
Inconvenient	0 (0.0%)
Very inconvenient	0 (0.0%)
No response	0 (0.0%)
Environment satisfaction n (%)	
Very satisfied	163 (85.3%)
Satisfied	28 (14.7%)
Neutral	0 (0.0%)
Dissatisfied	0 (0.0%)
Very dissatisfied	0 (0.0%)
No response	0 (0.0%)

## Discussion

The RIO2 International study evaluated the safety of performing Reveal LINQ ICM insertions in-hospital, but outside the traditional procedure settings. It is the first study to characterize resource utilization, procedure time intervals, and physician satisfaction from multiple centers across different geographies, including Europe, Canada and Australia. All 191 insertion attempts were successful, and no untoward events were observed among the 176 patients evaluable for the primary endpoint during a follow-up period of 3 months. Additionally, there was high compliance with the protocol specified insertion procedure. Importantly, even under circumstances with deviations from protocol, there was no increase in the risk of adverse events, punctuating the benign nature of the insertion procedure. As such, these results provide strong evidence that the Reveal LINQ ICM insertion can be safely performed out-of-lab within the hospital by following good sterile technique and the manufacturer’s instructions. Other studies have assessed procedure safety outside the traditional hospital settings. In the randomized Reveal LINQ In-Office 2 (RIO 2) study performed in the United States, low rates of untoward events were observed when device insertion occurred in an office outside the hospital (*n* = 251; 0.8%) or in the traditional hospital setting (*n* = 231; 0.9%), with no difference between groups [3]. Two observational studies (performed in Denmark and Australia) compared the safety of ICM insertions performed in a hospital procedure room vs. EP lab and also reported low rates of across insertion environments (0.6–1.7%), with no significant differences between settings [4, 5]. Absent or low complication rates have been observed in three single arm studies in which all device insertions occurred in either a hospital holding area or procedure room (0.0–0.8%) [6–8]. Importantly, the above findings demonstrate similar or lower complication rates than were observed in a combined analysis of the Reveal LINQ Usability and Reveal LINQ Registry studies (0.7 and 1.6%, respectively), where most device insertions occurred in a cath/EP lab [9]. Finally, a recent analysis of the multi-center Reveal LINQ Registry in a larger population (*N* = 1222) confirmed these observations, with 0.5% vs 1.0% of complications occurring in patients with procedures performed outside of the EP/cath lab vs in the EP/cath lab, respectively [10]. Together, these findings suggest that insertion of miniaturized ICMs can be moved outside the traditional hospital settings without compromising patient safety.

It is notable that no infections were observed in the present investigation, even under circumstances requiring device repositioning and despite pre-procedure antibiotics only being administered in 44% of patients. This corresponds with previous studies reporting low infection rates across insertion environments regardless of antibiotic use. Specifically, no infections were observed when an ICM was inserted in either the office (*n* = 251) or traditional hospital setting (*n* = 231) in the randomized RIO 2 US study. In that investigation, prophylactic antibiotics were administered to approximately 45% of patients, and the rate did not differ between groups [3]. In addition, the Reveal LINQ Registry showed an infection rate of 0.7% among patients who had the procedure performed out-of-lab, and 0.5% for those located in-lab, with similar infection rates observed in patients irrespective of prophylactic antibiotic administration [10]. An interim analysis of the randomized LOOP trial did demonstrate a higher rate of infections following device insertion in a procedure room vs. EP lab (1.6% vs. 0.1%, *p* = 0.004) despite administration of pre-procedural antibiotics in over 95% of patients [4]. However, the infection rate in the procedure room remained low and comparable to rates observed in-lab in other studies [9, 10]. LOOP also showed a trend for fewer adverse events, including infections, with an increase in physician experience. Lastly, two single arm studies where device insertion occurred in a hospital holding area or procedure room observed infection rates of 0.0 and 0.8% when prophylactic antibiotics were used in none and all insertion procedures, respectively [6, 7]. Together, these data suggest that infection rates with miniaturized ICM insertion are low (≤1.6%) across insertion environments irrespective of antibiotic prophylaxis.

One of the novelties in the present investigation was the characterization of time intervals during the different steps of the patient care pathway across multiple centers and geographies (patient preparation, procedure room occupancy, process time, education, and total visit duration). We observed that device insertion could be completed efficiently out-of-lab with an average visit duration of 115 min, procedure room occupancy time of 54 min, and process duration of 29 min (from patient preparation to skin closure). Others have reported on room occupancy times and have found similar results (55–58 min) [2, 11]. Moreover, in the present study physicians reported few delays over 15 min. This corresponds with findings from the RIO 2 US study, where physicians reported fewer delays when a miniaturized ICM was inserted in an office vs. in-lab [3]. Together, these observations suggest that moving device insertion outside the traditional hospital settings can result in time savings.

Another interesting finding was the involvement of non-physician personnel throughout the care pathway. Although physicians participated in most device insertions, registered nurses and cardiovascular/surgical technologists were predominant in performing pre- and post-insertion activities, respectively. Other single-center studies have also described successful experiences with non-physician providers inserting miniaturized ICMs in ambulatory settings in terms of safety, improved patient flow, reduced costs, and better allocation of resources [6, 11, 12].

Finally, physician perception of performing the procedure outside the traditional settings was very positive in terms of satisfaction with the environment, convenience, and positive feedback from patients. Results from the RIO 2 randomized study, comparing Reveal LINQ insertions performed in-office vs in the traditional hospital settings, also showed improved patient and physician experience with in-office insertions [3].

### Limitations

The main limitation of the study is its nonrandomized and single arm nature. However, safety of in-lab device insertion has been well established in previous investigations. Another limitation is the fact that the sample size was lower than estimated (191 vs. 204). In addition, the primary endpoint could not be ascertained in 15 patients due to early study withdrawal. However, patients were free from adverse events at the time of study withdrawal and the low untoward rate observed in those who were assessable would indicate that these patients were highly unlikely to have experienced an untoward event. Lastly, the study did not include a standardized approach to collect costs, so the hospital cost burden could not be estimated.

## Conclusions

Findings from the RIO 2 International study, the first prospective, international, multicenter trial of its kind, add to a growing body of evidence that the Reveal LINQ ICM can be safely and efficiently inserted outside the traditional hospital settings.

## Additional file

Additional file 1: Physician Questionnaire. (PDF 89 kb)

## Data Availability

The datasets generated and/or analyzed during the current study are not publicly available because the data that is used is confidential, but they are available from the corresponding author on reasonable request.

## Abbreviations

AE: Adverse event
Cath lab: Cardiac catheterization laboratory
CV/OR technologist: Cardiovascular/surgical technologist
EP lab: Electrophysiology laboratory
ICM: Insertable cardiac monitor
In-lab: Inside a cardiac catheterization laboratory, electrophysiology laboratory or operating room
IQR: Interquartile range
OR: Operating room
Out-of-lab: Outside a cardiac catheterization laboratory, electrophysiology laboratory or operating room
RIO 2: Reveal LINQ™ in-office 2

## References

[1] Tomson TT, Passman R (2015). The reveal LINQ insertable cardiac monitor. Expert Rev Med Devices.

[2] Kanters TA, Wolff C, Boyson D, Kouakam C, Dinh T, Hakkaart L, Rutten-Van Molken MP (2016). Cost comparison of two implantable cardiac monitors in two different settings: reveal XT in a catheterization laboratory vs. reveal LINQ in a procedure room. Europace : European pacing, arrhythmias, and cardiac electrophysiology : journal of the working groups on cardiac pacing, arrhythmias, and cardiac cellular electrophysiology of the European Society of Cardiology.

[3] Rogers JD, Sanders P, Piorkowski C, Sohail MR, Anand R, Crossen K, Khairallah FS, Kaplon RE, Stromberg K, Kowal RC (2017). In-office insertion of a miniaturized insertable cardiac monitor: results from the reveal LINQ in-office 2 randomized study. Heart Rhythm.

[4] Diederichsen SZ, Haugan KJ, Hojberg S, Holst AG, Kober L, Pedersen KB, Graff C, Krieger D, Brandes A, Svendsen JH (2017). Complications after implantation of a new-generation insertable cardiac monitor: results from the LOOP study. Int J Cardiol.

[5] Wong GR, Lau DH, Middeldorp ME, Harrington JA, Stolcman S, Wilson L, Twomey DJ, Kumar S, Munawar DA, Khokhar KB (2016). Feasibility and safety of reveal LINQ insertion in a sterile procedure room versus electrophysiology laboratory. Int J Cardiol.

[6] Kipp R, Young N, Barnett A, Kopp D, Leal MA, Eckhardt LL, Teelin T, Hoffmayer KS, Wright J, Field M (2017). Injectable loop recorder implantation in an ambulatory setting by advanced practice providers: analysis of outcomes. Pacing Clin Electrophysiol.

[7] Maines M, Zorzi A, Tomasi G, Angheben C, Catanzariti D, Piffer L, Del Greco M (2017). Clinical impact, safety, and accuracy of the remotely monitored implantable loop recorder Medtronic reveal LINQ™. Europace : European pacing, arrhythmias, and cardiac electrophysiology : journal of the working groups on cardiac pacing, arrhythmias, and cardiac cellular electrophysiology of the European Society of Cardiology.

[8] Lee JJ, Weitz D, Anand R (2017). Holding area LINQ trial (HALT). Indian Pacing Electrophysiol J.

[9] Mittal S, Sanders P, Pokushalov E, Dekker L, Kereiakes D, Schloss EJ, Pouliot E, Franco N, Zhong Y, DI Bacco M (2015). Safety profile of a miniaturized Insertable cardiac monitor: results from two prospective trials. Pacing Clin Electrophysiol.

[10] Beinart SC, Natale A, Verma A, Amin A, Kasner S, Diener HC, Del Greco M, Wilkoff BL, Pouliot E, Franco N (2019). Real-world comparison of in-hospital reveal LINQ insertable cardiac monitor insertion inside and outside of the cardiac catheterization or electrophysiology laboratory. Am Heart J.

[11] Di Odoardo LAF, Ambrosini F, Giavarini A, Vicenzi M, Venturini F, Lombardi F (2017). Reveal LINQTM experience out of the electrophysiology lab. J Cardiovasc Med (Hagerstown).

[12] Roebuck A, Mercer C, Denman J, Houghton AR, Richard A (2015). Experiences from a non-medical, non-catheter laboratory implantable loop recorder (ILR) service. Br J Cardiol.

