# Supplementary material for: Safety of in-hospital insertable cardiac monitor procedures performed outside the traditional settings: results from the Reveal LINQ in-office 2 international study
Source: BMC Cardiovasc Disord. 2019 May 31;19:132. doi: 10.1186/s12872-019-1106-3 (PMC6545016; doi:10.1186/s12872-019-1106-3)
Supplement: Supplementary file 1 — Physician Questionnaire. (PDF 89 kb) [file 12872_2019_1106_MOESM1_ESM.pdf]

Physician Questionnaire

Physicians were asked the following questions after each ICM insertion procedure:

1. How well did your Subject respond to having their Reveal LINQ™ insertion procedure today?
2. Was the procedure delayed past its scheduled start time by more than 15 minutes?
3. Estimate of your physician work time related to pre-service period for this subject
4. Estimate of your physician work time related to post-service period for this subject
5. Please rate your convenience of using this facility today for the Reveal LINQ™ procedure, compared to your standard facility.
6. How satisfied were you with performing this procedure in this environment of care?
